# Redetermination of the crystal structure of β-zinc molybdate from single-crystal X-ray diffraction data

**DOI:** 10.1107/S205698901501186X

**Published:** 2015-06-27

**Authors:** Olfa Mtioui-Sghaier, Rafael Mendoza-Meroño, Lilia Ktari, Mohamed Dammak, Santiago García-Granda

**Affiliations:** a Departamento de Química Física y Analítica, Facultad de Química, Universidad de Oviedo-CINN, C/ Julián Clavería, 8, 33006 Oviedo, Spain; b Laboratoire de Chimie Inorganique, Faculté des Sciences de Sfax, Route de Soukra, 3000 Sfax, Tunisia

**Keywords:** crystal structure, redetermination, β-ZnMoO_4_, hydro­thermal synthesis, wolframite structure type

## Abstract

The crystal structure of the β-polymorph of ZnMoO_4_ was re-determined on the basis of single-crystal X-ray diffraction data. In comparison with previous powder X-ray diffraction studies [Katikaneani & Arunachalam (2005 ▸). *Eur. J. Inorg. Chem*. pp. 3080–3087; Cavalcante *et al.* (2013 ▸). *Polyhedron*, **54**, 13–25], all atoms were refined with anisotropic displacement parameters, leading to a higher precision with respect to bond lengths and angles. β-ZnMoO_4_ adopts the wolframite structure type and is composed of distorted ZnO_6_ and MoO_6_ octa­hedra, both with point group symmetry 2. The distortion of the octa­hedra is reflected by variation of bond lengths and angles from 2.002 (3)–2.274 (4) Å, 80.63 (11)–108.8 (2)° for equatorial and 158.4 (2)– 162.81 (14)° for axial angles (ZnO_6_), and of 1.769 (3)–2.171 (3) Å, 73.39 (16)–104.7 (2), 150.8 (2)–164.89 (15)° (MoO_6_), respectively. In the crystal structure, the same type of *M*O_6_ octa­hedra share edges to built up zigzag chains extending parallel to [001]. The two types of chains are condensed by common vertices into a framework structure. The crystal structure can alternatively be described as derived from a distorted hexa­gonally closed packed arrangement of the O atoms, with Zn and Mo in half of the octa­hedral voids.

## Related literature   

Most molybdates of divalent cations crystallize either in the scheelite-type or in the wolframite-type (Macavei & Schulz, 1993 ▸). Zinc molybdate (ZnMoO_4_) is an inorganic semiconductor. It adopts the wolframite-type of structure (Keeling, 1957 ▸) and is dimorphic. The two phases, referred to as α- (triclinc symmetry) and β- (monoclinic symmetry), can be selectively obtained by controlling the synthetic conditions (Abrahams *et al.*, 1967 ▸; Zhang *et al.*, 2010 ▸). Previous crystal structure refinements of β-ZnMoO_4_, based on X-ray powder diffraction data, were reported by Cavalcante *et al.* (2013 ▸) and Katikaneani & Arunachalam (2005 ▸). For structure refinement of ZnWO_4_, isotypic with the title compound, see: Trots *et al.* (2009 ▸).

## Experimental   

### Crystal data  

ZnMoO_4_
*M* *_r_* = 225.31
Monoclinic, 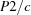

*a* = 4.6980 (3) Å
*b* = 5.7380 (4) Å
*c* = 4.8960 (4) Å
β = 90.311 (7)°
*V* = 131.98 (2) Å^3^
*Z* = 2
Mo *K*α radiation
μ = 13.62 mm^−1^
*T* = 293 K
0.08 × 0.06 × 0.03 mm

### Data collection  

Oxford Diffraction Xcalibur CCD diffractometer
Absorption correction: multi-scan (*CrysAlis PRO*; Oxford Diffraction, 2014 ▸) *T*_min_ = 0.905, *T*_max_ = 1.000
1207 measured reflections
405 independent reflections
358 reflections with *I* > 2σ(*I*)
*R*_int_ = 0.036

### Refinement  

*R*[*F* ^2^ > 2σ(*F* ^2^)] = 0.028
*wR*(*F* ^2^) = 0.068
*S* = 1.10
405 reflections
29 parameters
Δρ_max_ = 1.20 e Å^−3^
Δρ_min_ = −1.17 e Å^−3^

### 

Data collection: *CrysAlis CCD* (Oxford Diffraction, 2014 ▸); cell refinement: *CrysAlis RED* (Oxford Diffraction, 2014 ▸); data reduction: *CrysAlis RED*; program(s) used to solve structure: *SIR2011* (Burla *et al.*, 2012 ▸); program(s) used to refine structure: *SHELXL2014* (Sheldrick, 2015 ▸); molecular graphics: *DIAMOND* (Brandenburg & Putz, 1999 ▸); software used to prepare material for publication: *WinGX* (Farrugia, 2012 ▸), *publCIF* (Westrip, 2010 ▸) and *PARST* (Nardelli, 1995 ▸).

## Supplementary Material

Crystal structure: contains datablock(s) I, New_Global_Publ_Block. DOI: 10.1107/S205698901501186X/wm5159sup1.cif

Structure factors: contains datablock(s) I. DOI: 10.1107/S205698901501186X/wm5159Isup2.hkl

**Fig. 1.**
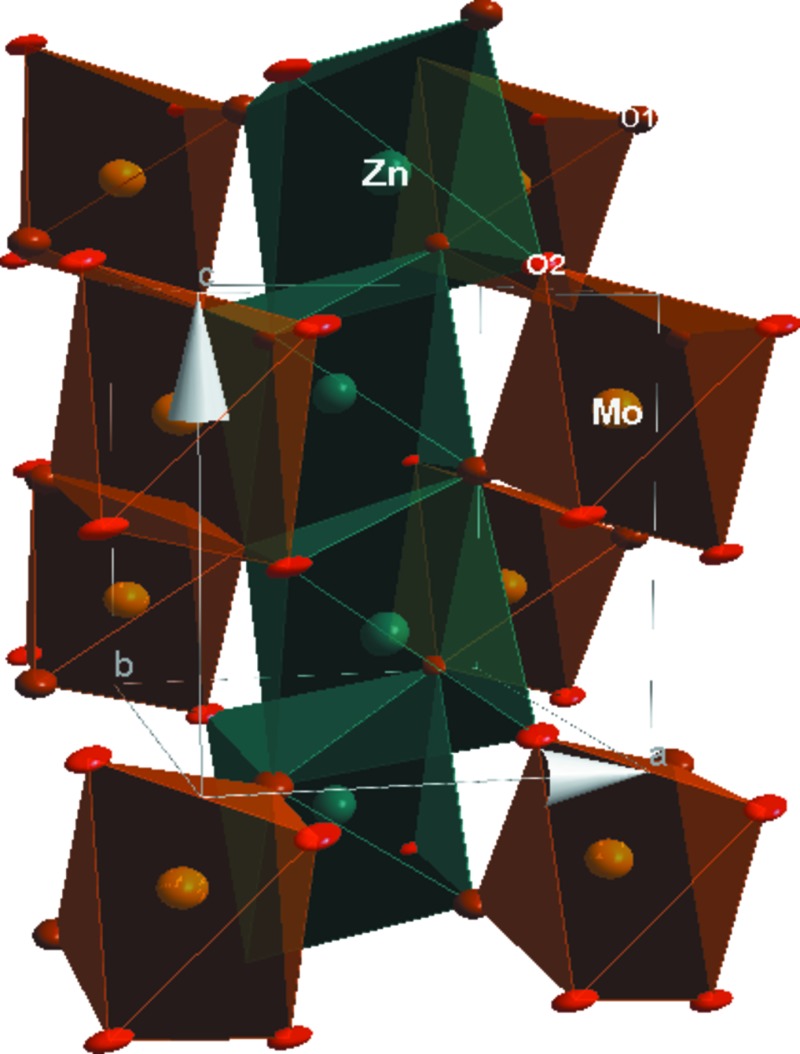
A view of the crystal structure of β-ZnMoO4. Anisotropic displacement parameters are drawn at the 50% probability level.

4 . DOI: 10.1107/S205698901501186X/wm5159fig1.tif
A view of the crystal structure of β-ZnMoO_4_. Anisotropic displacement parameters are drawn at the 50% probability level.

CCDC reference: 1408028

Additional supporting information:  crystallographic information; 3D view; checkCIF report

## supplementary crystallographic information

### S1. Synthesis and crystallization

Reagents were used as commercial sources with no further purification. An aqueous solution was prepared by a mixture of 0.047 g 2,2'-bi­pyridine, 0.015 g of molybdenum trioxide and 0.043 g of zinc acetate in 10 ml water. The reaction mixture was stirred at room temperature to homogeneity, then transferred into a teflon-lined stainless steel vessel (40 ml) and heated to 453 K for 48 h under autogenous pressure and after-wards cooled slowly to room temperature. The resulting material was obtained as colorless single-crystals without side products. The solid was filtered off, washed thoroughly with distilled water, and finally air-dried at room temperature.

### S2. Refinement

The remaining maximum and minimum electron densities were found 0.77 Å and 0.90 Å, respectively, from the O1 atom. O1 is located on the center of a tree-metal triangle, bridging one Mo and two Zn atoms.

### Crystal data

**Table:**

MoO_4_Zn	*F*(000) = 208
*M**_r_* = 225.31	*D*_x_ = 5.670 Mg m^−^^3^
Monoclinic, *P*2/*c*	Mo *K*α radiation, λ = 0.71073 Å
*a* = 4.6980 (3) Å	Cell parameters from 570 reflections
*b* = 5.7380 (4) Å	θ = 3.6–31.1°
*c* = 4.8960 (4) Å	µ = 13.62 mm^−^^1^
β = 90.311 (7)°	*T* = 293 K
*V* = 131.98 (2) Å^3^	Prism, colourless
*Z* = 2	0.08 × 0.06 × 0.03 mm

### Data collection

**Table:**

Oxford Diffraction Xcalibur CCD diffractometer	405 independent reflections
Radiation source: Enhance (Mo) X-ray Source	358 reflections with *I* > 2σ(*I*)
Detector resolution: 10.2673 pixels mm^-1^	*R*_int_ = 0.036
ω– and φ–scans	θ_max_ = 31.3°, θ_min_ = 3.6°
Absorption correction: multi-scan (*CrysAlis PRO*; Oxford Diffraction, 2014)	*h* = −6→6
*T*_min_ = 0.905, *T*_max_ = 1.000	*k* = −8→8
1207 measured reflections	*l* = −6→6

### Refinement

**Table:**

Refinement on *F*^2^	29 parameters
Least-squares matrix: full	0 restraints
*R*[*F*^2^ > 2σ(*F*^2^)] = 0.028	*w* = 1/[σ^2^(*F*_o_^2^) + (0.0209*P*)^2^ + 0.5403*P*] where *P* = (*F*_o_^2^ + 2*F*_c_^2^)/3
*wR*(*F*^2^) = 0.068	(Δ/σ)_max_ < 0.001
*S* = 1.10	Δρ_max_ = 1.20 e Å^−^^3^
405 reflections	Δρ_min_ = −1.17 e Å^−^^3^

### Special details

**Table:**

Geometry. All e.s.d.'s (except the e.s.d. in the dihedral angle between two l.s. planes) are estimated using the full covariance matrix. The cell e.s.d.'s are taken into account individually in the estimation of e.s.d.'s in distances, angles and torsion angles; correlations between e.s.d.'s in cell parameters are only used when they are defined by crystal symmetry. An approximate (isotropic) treatment of cell e.s.d.'s is used for estimating e.s.d.'s involving l.s. planes.

### Fractional atomic coordinates and isotropic or equivalent isotropic displacement parameters (Å^2^)

**Table:**

	*x*	*y*	*z*	*U*_iso_*/*U*_eq_	
Mo1	1.0000	0.81190 (10)	0.2500	0.00507 (18)	
Zn1	1.5000	0.69182 (15)	0.7500	0.0092 (2)	
O1	1.2538 (7)	0.6236 (6)	0.4014 (7)	0.0080 (7)	
O2	0.7835 (7)	0.8950 (6)	0.5603 (7)	0.0058 (7)	

### Atomic displacement parameters (Å^2^)

**Table:**

	*U*^11^	*U*^22^	*U*^33^	*U*^12^	*U*^13^	*U*^23^
Mo1	0.0065 (3)	0.0045 (3)	0.0041 (3)	0.000	−0.0002 (2)	0.000
Zn1	0.0087 (4)	0.0116 (4)	0.0073 (4)	0.000	0.0009 (3)	0.000
O1	0.0089 (17)	0.0110 (16)	0.0041 (17)	0.0006 (14)	0.0003 (13)	−0.0004 (14)
O2	0.0088 (16)	0.0064 (14)	0.0021 (16)	−0.0005 (13)	0.0016 (12)	−0.0010 (13)

### Geometric parameters (Å, º)

**Table:**

Mo1—O1^i^	1.769 (3)	Zn1—O2^iv^	2.002 (3)
Mo1—O1	1.769 (3)	Zn1—O2^v^	2.002 (3)
Mo1—O2	1.894 (3)	Zn1—O1^vi^	2.094 (3)
Mo1—O2^i^	1.894 (3)	Zn1—O1	2.094 (3)
Mo1—O2^ii^	2.171 (3)	Zn1—O1^vii^	2.274 (4)
Mo1—O2^iii^	2.171 (3)	Zn1—O1^viii^	2.274 (4)
			
O1^i^—Mo1—O1	104.7 (2)	O2^iv^—Zn1—O1	95.54 (14)
O1^i^—Mo1—O2	97.25 (15)	O2^v^—Zn1—O1	96.96 (14)
O1—Mo1—O2	100.46 (15)	O1^vi^—Zn1—O1	158.4 (2)
O1^i^—Mo1—O2^i^	100.46 (15)	O2^iv^—Zn1—O1^vii^	162.81 (14)
O1—Mo1—O2^i^	97.25 (15)	O2^v^—Zn1—O1^vii^	88.37 (13)
O2—Mo1—O2^i^	150.8 (2)	O1^vi^—Zn1—O1^vii^	82.25 (14)
O1^i^—Mo1—O2^ii^	164.89 (14)	O1—Zn1—O1^vii^	80.63 (11)
O1—Mo1—O2^ii^	88.90 (14)	O2^iv^—Zn1—O1^viii^	88.37 (13)
O2—Mo1—O2^ii^	73.39 (16)	O2^v^—Zn1—O1^viii^	162.81 (14)
O2^i^—Mo1—O2^ii^	84.01 (11)	O1^vi^—Zn1—O1^viii^	80.63 (11)
O1^i^—Mo1—O2^iii^	88.90 (14)	O1—Zn1—O1^viii^	82.24 (14)
O1—Mo1—O2^iii^	164.89 (15)	O1^vii^—Zn1—O1^viii^	74.53 (18)
O2—Mo1—O2^iii^	84.01 (11)	Mo1—O1—Zn1	126.54 (19)
O2^i^—Mo1—O2^iii^	73.39 (16)	Mo1—O1—Zn1^viii^	133.83 (18)
O2^ii^—Mo1—O2^iii^	78.49 (18)	Zn1—O1—Zn1^viii^	97.75 (14)
O2^iv^—Zn1—O2^v^	108.8 (2)	Mo1—O2—Zn1^ix^	125.93 (18)
O2^iv^—Zn1—O1^vi^	96.96 (14)	Mo1—O2—Mo1^ii^	106.61 (16)
O2^v^—Zn1—O1^vi^	95.54 (14)	Zn1^ix^—O2—Mo1^ii^	124.32 (17)

Symmetry codes: (i) −*x*+2, *y*, −*z*+1/2; (ii) −*x*+2, −*y*+2, −*z*+1; (iii) *x*, −*y*+2, *z*−1/2; (iv) *x*+1, *y*, *z*; (v) −*x*+2, *y*, −*z*+3/2; (vi) −*x*+3, *y*, −*z*+3/2; (vii) *x*, −*y*+1, *z*+1/2; (viii) −*x*+3, −*y*+1, −*z*+1; (ix) *x*−1, *y*, *z*.
