# Supplementary figures and images for: Redetermination of the crystal structure of β-zinc molybdate from single-crystal X-ray diffraction data
Source: Acta Crystallogr E Crystallogr Commun. 2015 Jun 27;71(Pt 7):i6–7. doi: 10.1107/S205698901501186X (PMC4518998; doi:10.1107/S205698901501186X)

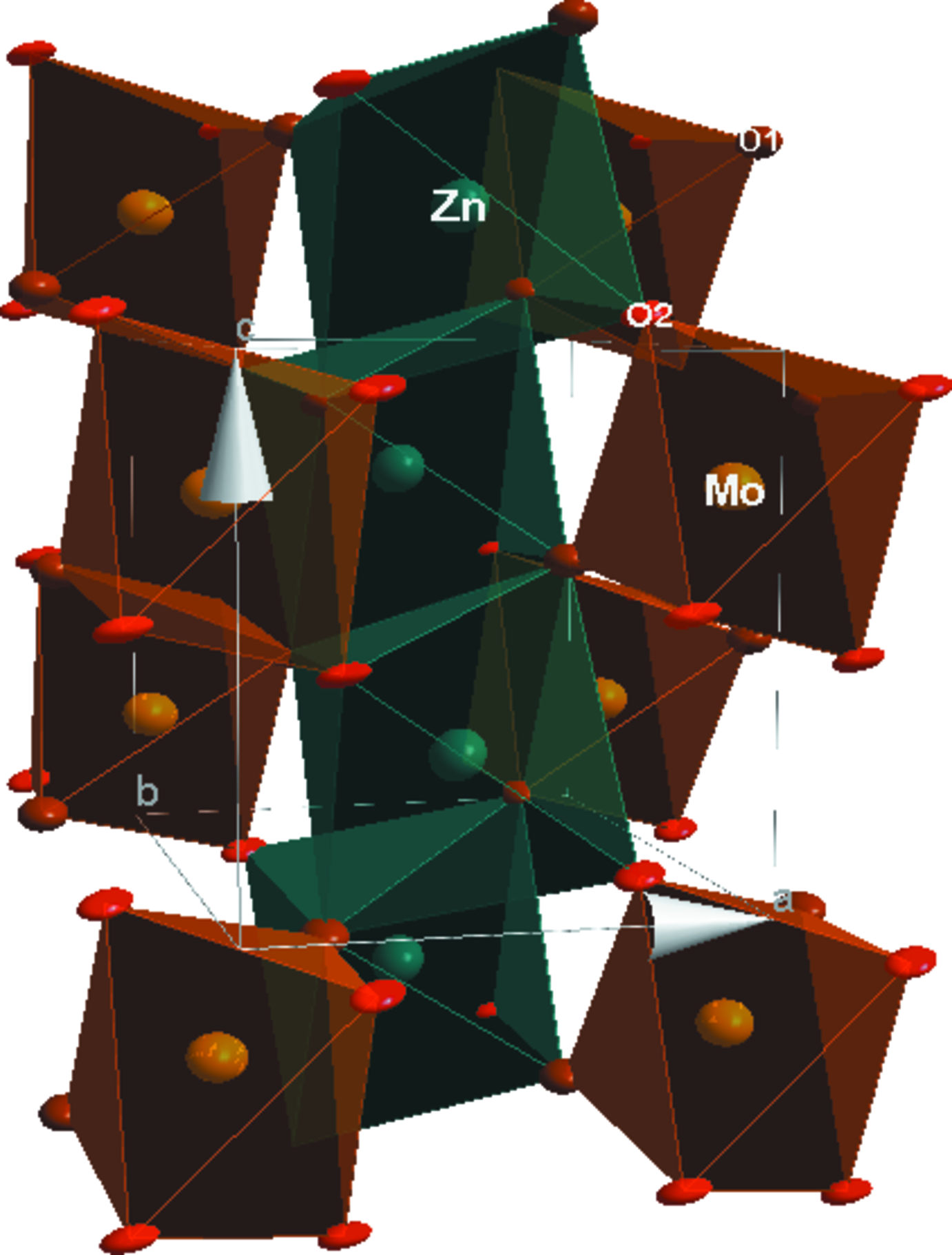

Supplement: Supplementary file 3 [file e-71-000i6-fig1.tif]
